# Effects of Brush-Type Ionizer Materials on Virus Inactivation

**DOI:** 10.3390/toxics10100611

**Published:** 2022-10-14

**Authors:** Jaeseok Heo, Jooyeon Lee, Duckshin Park

**Affiliations:** 1 Transportation Environmental Research Department, Korea Railroad Research Institute, Uiwang 16105, Korea; 2 Railway System Engineering, University of Science and Technology, Daejeon 34113, Korea

**Keywords:** ionizer, bioaerosols, brush, inactivation, flow velocity

## Abstract

Many studies have found that bioaerosols are harmful to humans. In particular, infectious viruses, such as the virus that causes COVID-19, are increasing. Therefore, the research on methods for reducing bioaerosols is becoming progressively more important. The purpose of this study was to improve the existing electrostatic precipitator, which generates high concentrations of ozone, by reducing bioaerosols effectively without significant ozone production. A brush-type ionizer was studied as a replacement for the existing electrostatic precipitator. The study, which was conducted at the laboratory scale, determined the amounts of ions generated with different ionizer materials (carbon, copper, and stainless steel) and voltages (−1, −2, and −3 kV), as well as it compared the virus inactivation efficiency under the various conditions. As a result, about two million ions were produced when a voltage of −3 kV was applied to all of the materials, and 99.9 ± 0.2% and 98.8 ± 0.6% virus inactivation efficiencies were confirmed in the cases of carbon and copper, respectively. In addition, an assessment of the effect of flow velocity confirmed that the inactivation efficiency decreased as the flow velocity increased. However, the results for the flow velocities of 0.2 and 0.4 m/s had similar trends. Therefore, this system can be used with flow velocities up to 0.4 m/s.

## 1. Introduction

Bioaerosols, which are harmful to humans [1,2], are artificially produced or naturally occurring substances of biological origin, including bacteria, fungi, viruses, and animal dander [3]. Problems associated with bioaerosols arise frequently. In particular, the interest in viruses has increased recently due to COVID-19, a new viral disease that is transmitted through droplets. COVID-19, which is highly contagious, has spread throughout the world, and the World Health Organization (WHO) declared it a pandemic on 11 March 2020 [4]. Viruses are only nanometers in size, ranging from 20–400 nm, and they can remain in the air for long periods of time [2,5]. Viruses have a low sedimentation rate and survive well in the air depending on factors such as absolute humidity and the physical and chemical properties of the droplets [6,7,8]. There are various methods for reducing these harmful particles in the air, such as the filtration method and the dust collection method [2,9]. Since the filtration method does not directly eliminate bioaerosols, which can cause microorganisms to reproduce on the filter and substances such as microbial volatile organic compounds to be generated [9,10]. Thus, the problem of secondary pollutants arises [9]. Research pertaining to the electrical dust collection method has mainly been conducted with the goal of generating an artificial electrostatic force by applying a high voltage using a corona discharge charging system before and after the filter [11,12]. However, the corona discharge used in the operation of the electrostatic precipitator may result in a gas that is harmful to the human body at high concentrations, such as the ozone generated in the process of charging particles, which may irritate the respiratory tract [12,13,14,15,16]. Accordingly, the Ministry of Environment recommended that the indoor ozone concentration in multi-use facilities, such as underground stations and waiting rooms, should be maintained below 0.06 ppm as of 2017. Previous studies have shown that the amount of ozone increases with the increase in the voltage and the diameter of the discharge electrode [15,17].

As mentioned, electrostatic precipitators generally generate ions through a corona discharge, and the ions induce charges on the particles, which are removed by a collection plate [18]. However, the corona discharge produces ozone [19].

In this study, the ozone generation was reduced by using a brush-type ionizer using carbon, copper, and stainless steel materials. Han et al. (2008) used carbon fiber to reduce the ozone and reported that ozone was not generated with applied voltages of up to −4 kV [18,20]. Ionization occurs when an electrically neutral atom or molecule acquires a positive or a negative charge when the available energy is excessive [21]. Previous studies have shown that negative ions, depending on their electrical characteristics, are effective at reducing microorganisms or fine dust [21,22,23]. Grinshpun et al. (2005), Daniels (2002), and Kondrashova et al. (2000) experimentally examined the removal effect of negative ions on contaminants, such as microorganisms [22,23,24]. Lee et al. (2022) revealed that the dust collection efficiency increased as the voltage of the ionizer was increased from −2.5 to −5 kV, but the ozone also increased [18]. When the voltage applied to the ionizer is increased, the corona discharge increases; thus, the number of charges per particle also increases [25]. As a result, the particle removal efficiency of the ionizer is enhanced [18]. Although the efficiency is improved when the voltage is increased, the ozone also increases, so it is necessary to determine the proper operating voltage of the ionizer. Therefore, to confirm the virus inactivation performance of the brush-type ionizer, the virus inactivation performance with each material and each voltage was studied at the laboratory scale to determine the optimal conditions.

## 2. Materials and Methods

### 2.1. Materials and Ion Measurement Experiments

**Figure 1:**
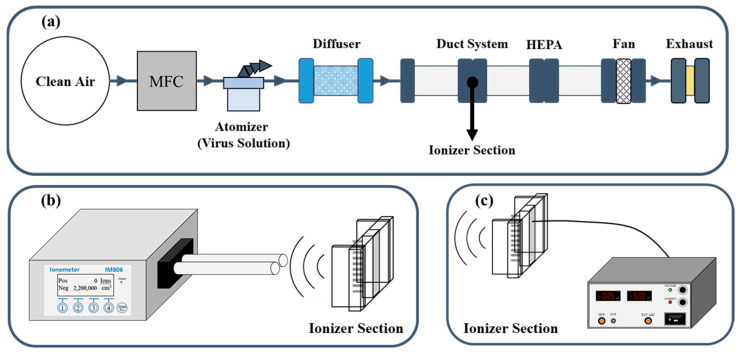
Duct system used for the measurements. (**a**) Duct system for the virus spread; (**b**) Measuring device setup for the ion measurement of the ionizers in the ducts; (**c**) Experimental concept to connect the ionizer and the voltage generator in the duct.

The corona discharge method, which is one of the discharge methods in gas generated around needle-shaped electrodes, was used to generate the ions. In this study, a brush-type ionizer capable of discharging at a low voltage was studied to solve the problem of the high concentration of ozone generated when using the pin-type discharge electrode, which has mainly been used for corona discharge. The generation of ions with the ionizer was assessed with carbon, copper, and stainless steel materials. An ionmeter (IM806, Holbach Umweltanalytik GmbH, Wadern, Germany) was used to measure the ions. After placing the ionizer in the duct system for measurements, the ions generated, according to the voltage and the materials used, were measured, as shown in Figure 1. Additionally, in order to check whether ozone was generated during the operation of the ionizer, the ozone was measured by using an Ozone Analyzer (49i, ThermoFisher Scientific, Franklin, PA, USA) and by inserting an inlet into the rear end of the ionizer in the duct.

### 2.2. Virus and Host Preparation

The virus used for this experiment was the MS2 bacteriophage (*Escherichia coli* bacteriophage MS2, American Type Culture Collection [ATCC] 15597-B1; ATCC, Manassas, VA, USA). Unlike the viruses that are harmful to the human body, this virus is non-pathogenic and has been used in several studies [5,8,26,27,28,29]. The virus was prepared by mixing a stock solution (100 μL) in 50 mL of sterile distilled water. The MS2 used in this experiment uses *E. coli* (*Escherichia coli*, ATCC 15597) as a host. This host was used after pre-culturing. A stock solution of *E. coli* (1000 μL) was prepared with 40 mL of sterilized trypticase soy broth. This mixture was incubated with shaking at 37 °C for 24 h.

### 2.3. Spray Aerosolized MS2 Bacteriophage

The virus was sprayed for 20 min using a single jet atomizer (model 9302, TSI Inc., Shoreview, MN, USA), generating particles at concentrations in excess of 10^7^ particles/cm^3^ at a nominal flow rate of 6.5 L/min. At this time, the pressure ranged from 180–200 kPa. The virus injection was carried out in the duct system. As shown in Figure 1, compressed air was pumped, cleaned, and controlled by a mass flow controller (MFC), and then it was connected to an atomizer and aerosolized. The virus solution was passed through the attached 4 × 4 duct, and the distance from the inlet to the ionizer was 60 cm [5]. A HEPA filter (H13, 99.965%, Geo Pure Tech, Seoul, Korea) was used as a control filter, as Mittal et al. found that it survived for 6 days without significant loss of viability upon contact with an MS2 bacteriophage [8,30]. All of the filter samples that collected viruses for each condition were randomly cut into pieces with a diameter of 1 cm, after which they were eluted in a U-APBS (10 mL of D.I., 0.9 mL of urea, 0.4 mL of 0.2 M phosphate buffer solution, and 0.4 mL of 0.5 M l-arginine solution) solution for 10 min. Approximately 0.1 mL of the soaked filter was transferred to dilute it to 1 mL, and serial dilutions were prepared.

### 2.4. Virus Inactivation According to Voltage

Lee et al. (2022) revealed that the dust collection efficiency increased as the voltage was increased from −4 kV to −5.5 kV due to the greater number of ions generated by the ionizer; however, ozone also increased to 7.8 ppb [18]. Moreover, Han et al. (2008) found that ozone was not generated at voltages of up to −4 kV when carbon fiber was used [20]. Therefore, based on previous work, the voltage was varied to confirm the performance without ozone generation at a low voltage. The materials of the brush-type ionizer each have their own threshold; thus, the corona discharge occurs above a certain voltage. Since the breakdown voltage of each material differs, a comparative experiment of virus inactivation according to voltage was conducted. To compare the virus inactivation for each material, the high voltage generator (B.125, Korea Switching, Seoul, Korea) that is connected to the ionizer and inserted into the duct was checked by varying its value to −1, −2, or −3 kV, as shown in Figure 1c.

### 2.5. Virus Inactivation According to Flow Velocity

In environments requiring indoor air quality, it is difficult to keep the flow velocity constant because products such as air conditioners and fans are used. In particular, the air flow rate inside the air conditioning system in subway stations is highly variable due to dampers [18]. Therefore, it is necessary to check the performance change accompanying the change in flow velocity with consideration given to fluidity. The study by Lee et al. found that the dust collection efficiency decreased as the flow velocity increased [18]. Thus, to confirm the proper use environment of the system, the flow velocity was varied and the performance was assessed. To confirm the virus inactivation according to the flow velocity, the experiment was conducted at wind speeds of 0.2, 0.4, and 0.6 m/s.

The virus inactivation was compared to the varying flow velocity by controlling the speed of the fan in the duct. The flow velocity in the duct was measured using an anemometer (Testo 405i, Testo Inc., Lenzkirch, Germany).

### 2.6. Calculation of Inactivation Efficiency

Plaque counts were performed to assess the results of the experiments. Approximately 100 μL of diluted solution of the control and the experimental comparison groups was mixed with the pre-cultured host (300 μL) and added to 29 mL of soft agar (trypticase soy agar, TSA). The mixture was placed in a petri dish with a diameter of 150 mm and incubated overnight at 37 °C. The control and ionizer-treated samples were compared according to the number of plaque-forming units per milliliter (PFU/mL). The equation used to calculate the PFU/mL number is as follows:

(1) $$\;N_{\left(PFU/mL\right)}=\frac{PFU}{\alpha }\times \frac{1}{d_{i}}\;,\;$$

where N is the number of PFU/mL, PFU is the total number of plaques (for all of the samples per one experiment), α is the volume of virus particles added (1 mL), and *d_i_* is the dilution factor. The mean plaque number was calculated and expressed in logarithmic form. The difference between the log values of the control and the ionizer-treated samples was calculated as follows:

(2) $$\;Diff{\;}_{Log_{10\;N}}=Log_{10}\;\left(N_{Control}\right)-Log_{10}\;\left(N_{ionizer}\right)\;,\;$$

where *Diff_Log_*_10*N*_ is the difference between the logarithmic values of the number of plaques in the control and in the ionizer-treated samples. The virus inactivation efficiency of the ionizer was calculated as follows:

(3)  IE%= 1−NionizerNcontrol×100 , 

where *IE_(%)_* is the inactivation efficiency, *N_ionizer_* is the number of colony-forming units per milliliter of the ionizer, and *N_control_* is the number of colony-forming units per milliliter of the control group. This study was conducted entirely at room temperature (25 °C) and at a relative humidity of 50% or less; the sampling was performed three times. The experimental preparation and inactivation efficiency calculations in this study were based on previous studies by Versoza et al. [5,8,29].

## 3. Results and Discussion

### 3.1. Experimental Results of the Ion Measurement under Each Condition of Voltage and Material

**Figure 2:**
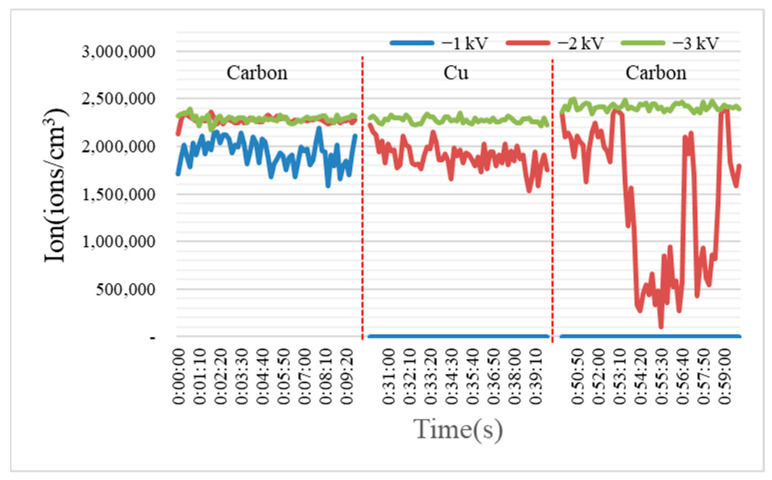
Ions generated by material and voltage.

Carbon, copper, and stainless steel were used as the brush materials for the ion generation. The comparison results of the amounts of ions generated with the different materials and voltages are shown in Figure 2.

The results of the ion amounts generated, classified by each material and voltage, showed that more than two million ions were generated at −3 kV. However, at −2 kV and −1 kV, the results differed depending on the material. In a previous study, Shin et al. found that the starting voltage was different for tungsten wire and carbon fiber wire with different diameters [31]. As described above, it seems that the amount of ions generated differs according to the voltage because the threshold value at which the discharge occurs is different for each material. The ion amounts generated with the different materials and voltages are listed in detail in Table 1. As shown in the Table 1, the deviation in the amount of ions is relatively small for carbon.

Hunt and Mariňas experimentally found that the concentration of ozone decreases the survival rate of microorganisms [32]. In addition, Kim et al. revealed that the ozone increased and then decreased with increasing the time during the operation of the ionizer, and that the ozone generated when the ionizer was operated for 10 min was 2.6 ppb [33]. In this study, while the virus was sprayed, the ionizer was operated and only measured for 20 min. At this time, about 1.6 ppb of ozone was generated at −3 kV with SUS, and the others showed insignificant changes. From this, it can be seen that the effect of ozone on virus inactivation was insignificant and that the inactivation was shown by the ions.

### 3.2. Experimental Results of the Virus Inactivation According to Voltage

**Figure 3:**
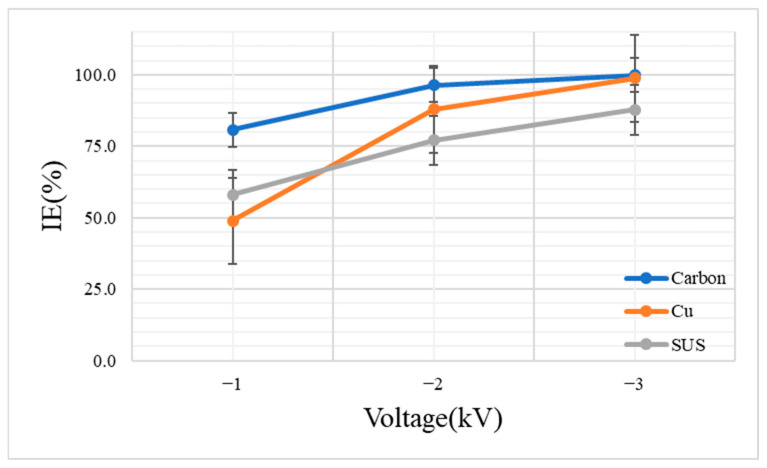
Inactivation efficiency (%) of the aerosolized MS2 bacteriophages in each material and at each voltage.

To confirm the effect of voltage on the MS2 bacteriophage inactivation, a solution containing 100 μL of the MS2 bacteriophage in 50 mL of sterilized deionized water was sprayed for 20 min. Figure 3 shows the MS2 bacteriophage inactivation efficiency for carbon, copper, and stainless steel for the conditions of −1, −2, and −3 kV.

As mentioned by Hyun et al. [2], the inactivation efficiency increased as the ion concentration increased. At −3 kV, which showed high ion generation, the high inactivation efficiencies of 99.9 ± 0.2%, 98.8 ± 0.6%, and 87.7 ± 2.3% were confirmed for carbon, copper, and stainless steel, respectively.

This result seems to be attributable to the stable generation of ions above the threshold voltage of each material. The result proved that the deactivation efficiency is directly proportional to the amount of ion generation. On the other hand, at −1 kV and −2 kV, the inactivation efficiencies were confirmed as follows: 80.8 ± 8.4% and 96.4 ± 0.5% for carbon; 48.9 ± 16.2% and 87.9 ± 6.2% for copper; 58.0 ± 2.3%, and 77.0 ± 3.4% for stainless steel. The amounts of ions generated by each material differed at low voltage, and the ion generation change was large in comparison to the case of −3 kV, which may have affected the inactivation efficiency.

Carbon showed higher inactivation efficiencies than copper or stainless steel at all voltages. In particular, at −3 kV, carbon had a significantly higher log10 PFU/mL value difference between the sample and the control compared with the other materials. The log10 PFU/mL value for carbon was 2.93; it was 1.93 for copper and 0.91 for stainless steel. This result indicates that all of the materials showed excellent inactivation performance above the threshold voltage in the case of the brush-type ionizer. Stainless steel had a lower deactivation efficiency than carbon or copper because of its relatively large ion deviation, but it has superior strength in comparison to the other materials. Additionally, stainless steel should perform as well as the other materials when used at a higher voltage. Therefore, stainless steel can be an alternative to other materials when there is a concern about durability. However, in the present study, carbon performed best under all conditions. The inactivation efficiency (%) and the logarithmic differences among carbon, copper, and stainless steel are shown in Table 2.

### 3.3. Experimental Results of the Virus Inactivation According to Flow Velocity

To check the deviation between the flow velocity in the duct and the flow velocity in the duct under each experimental condition, the flow velocity was measured for 20 min, which was the same as the virus injection time. As a result, the flow velocities of 0.2, 0.4, and 0.6 m/s were confirmed. The flow velocity was 0.2 m/s when the atomizer was used to spray the MS2 bacteriophage into the duct; it was approximately 0.4 and 0.6 m/s when a fan was used.

**Figure 4:**
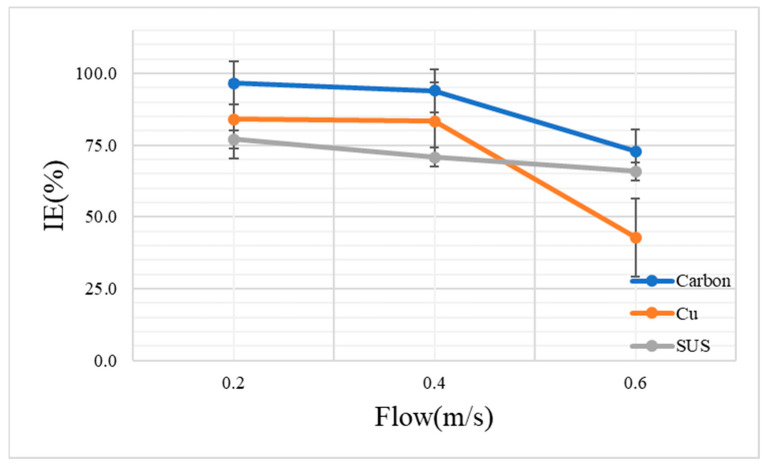
Inactivation efficiency (%) of the aerosolized MS2 bacteriophages for each material and flow velocity.

To confirm the effect of flow velocity on the MS2 bacteriophage inactivation, a solution containing 100 μL of the MS2 bacteriophage in 50 mL of sterilized deionized water was sprayed for 20 min at an applied ionizer voltage of −2 kV. Figure 4 shows the MS2 bacteriophage inactivation efficiency under the conditions of 0.2, 0.4, and 0.6 m/s when using carbon, copper, and stainless steel, respectively. In their previous work, Hyun et al. revealed that the antibacterial efficiency increased as the number of charges of ionic polarity increased and the flow velocity decreased [2]. Lee et al. found that the dust collection efficiency decreased as the flow velocity increased [18].

The results of the MS2 bacteriophage inactivation revealed that carbon, copper, and stainless steel had inactivation efficiencies of 96.6 ± 3.1%, 84.1 ± 7.4%, and 77.0 ± 3.4%, respectively, at a flow velocity of 0.2 m/s. As the flow velocity increased, the inactivation efficiency tended to decrease. In this experiment, the overall inactivation efficiency under the different flow velocities was rather low under the condition of −2 kV. However, the inactivation efficiency at the flow velocities of 0.2 and 0.4 m/s for carbon was 96.6 ± 3.1% and 94.1 ± 1.5%, respectively. This result indicates that carbon has stable inactivation efficiency up to a velocity of 0.4 m/s. On the other hand, copper showed the lowest inactivation efficiency of 42.9 ± 29.1% under the 0.6 m/s flow velocity condition. In particular, the inactivation efficiency of copper decreased sharply at 0.6 m/s in comparison to the efficiencies of the other materials. This result shows that copper is greatly affected by the flow velocity. This study compared the performance of brush-type ionizers from different materials. The system included no filters other than the ionizer. Therefore, because the structure of the ionizer was an open-type structure, if the flow velocity increased, the virus could pass before the generated ions, and the virus would come into contact. This result is consistent with the study result of Nunayon et al., which is that the increase in the flow velocity reduces the contact time of the bioaerosol exposed to the ion regardless of the polarity of the ion [34]. Therefore, this result clearly showed that the inactivation efficiency decreased when the flow velocity was increased. The inactivation efficiency (%) and the logarithmic differences among carbon, copper, and stainless steel under the condition of −2 kV are shown in Table 3.

## 4. Conclusions

Ozone generation can be a problem when existing electrostatic precipitators are used to ensure air quality. Restrictions on the use of pin-type electrostatic precipitators have been implemented because regulations were established to restrict ozone generation. For this reason, research towards ozone reduction is being carried out actively.

This study evaluated an air purification device capable of operating at a low voltage that can reduce fine dust and bioaerosols, as well as minimize ozone generation. This air purifier utilized a brush-type ionizer using corona discharge. The comparisons of the amounts of ions generated and the virus inactivation efficiency with various materials, voltages, and flow velocities led to the below conclusions.

First, the comparison of ion generation according to the material and voltage revealed that about 2.4 million ions were generated when a voltage of −3 kV was applied using a stainless steel material. However, stainless steel had a slightly larger ion deviation compared to the other materials. When the same voltage of −3 kV was applied with the carbon material, about 2.3 million ions were generated, which is slightly fewer than with stainless steel, but the ions were generated in a more stable fashion than with the stainless steel. In addition, the comparison of the virus inactivation efficiency under the same conditions revealed that a 99.9% efficiency was obtained with a voltage of −3 kV and carbon. The results demonstrated high inactivation efficiency when the ions were generated stably. Finally, the comparison showed that the inactivation efficiency decreased as the flow velocity increased. In the case of carbon, which had the highest efficiency, a relatively high inactivation efficiency of 94.1% was attained at a flow velocity of 0.4 m/s. Based on these results and under the conditions of this study, both carbon and copper can be used for the ionizer at a voltage of −3 kV and up to a flow velocity of 0.4 m/s.

This study evaluated a reduction device that can replace the electrostatic precipitator applied in air conditioning systems. However, as an experimental step, this study was conducted at the laboratory scale and excluded problems that could occur during field applications. Therefore, the scale of the research needs to be expanded to include field-applicable ionizer evaluations.

## Figures and Tables

**Table 1:** Average ion amount generated by material and voltage.

	−1 kV	−2 kV	−3 kV
Carbon	1,934,381 ± 1.4 × 10^5^	2,274,782 ± 0.3 × 10^5^	2,289,422 ± 0.3 × 10^5^
Copper	37 ± 20	1,893,961 ± 1.3 × 10^5^	2,276,150 ± 0.3 × 10^5^
Stainless steel	213 ± 21	1,428,998 ± 7.5 × 10^5^	2,413,693 ± 0.4 × 10^5^

**Table 2:** Inactivation efficiencies (%) and logarithmic differences for the aerosolized MS2 bacteriophages for each material and voltage.

Material	Voltage (kV)	IE (%)	Diff (_Log 10 PFU/mL_)
Carbon	−1	80.8 ± 8.4%	0.72
	−2	96.4 ± 0.5%	1.44
	−3	99.9 ± 0.2%	2.93
Copper	−1	48.9 ± 16.2%	0.29
	−2	87.9 ± 6.2%	0.92
	−3	98.8 ± 0.6%	1.93
Stainless steel	−1	58.0 ± 2.3%	0.38
	−2	77.0 ± 3.4%	0.64
	−3	87.7 ± 2.3%	0.91

**Table 3:** Inactivation efficiency (%) and logarithmic difference of the aerosolized MS2 bacteriophages for each material and flow velocity.

Voltage (kV)	Material	Flow Velocity (m/s)	IE (%)	Diff (_Log 10 PFU/mL_)
−2	Carbon	0.2	96.6 ± 3.1%	1.47
		0.4	94.1 ± 1.5%	1.23
		0.6	72.9 ± 22.1%	0.57
	Copper	0.2	84.1 ± 7.4%	0.80
		0.4	83.4 ± 5.0%	0.78
		0.6	42.9 ± 29.1%	0.24
	Stainless steel	0.2	77.0 ± 3.4%	0.64
		0.4	70.8 ± 2.2%	0.53
		0.6	65.8 ± 3.2%	0.47

## Data Availability

Not applicable.
